# The International Bathymetric Chart of the Southern Ocean Version 2

**DOI:** 10.1038/s41597-022-01366-7

**Published:** 2022-06-07

**Authors:** Boris Dorschel, Laura Hehemann, Sacha Viquerat, Fynn Warnke, Simon Dreutter, Yvonne Schulze Tenberge, Daniela Accettella, Lu An, Felipe Barrios, Evgenia Bazhenova, Jenny Black, Fernando Bohoyo, Craig Davey, Laura De Santis, Carlota Escutia Dotti, Alice C. Fremand, Peter T. Fretwell, Jenny A. Gales, Jinyao Gao, Luca Gasperini, Jamin S. Greenbaum, Jennifer Henderson Jencks, Kelly Hogan, Jong Kuk Hong, Martin Jakobsson, Laura Jensen, Johnathan Kool, Sergei Larin, Robert D. Larter, German Leitchenkov, Benoît Loubrieu, Kevin Mackay, Larry Mayer, Romain Millan, Mathieu Morlighem, Francisco Navidad, Frank O. Nitsche, Yoshifumi Nogi, Cécile Pertuisot, Alexandra L. Post, Hamish D. Pritchard, Autun Purser, Michele Rebesco, Eric Rignot, Jason L. Roberts, Marzia Rovere, Ivan Ryzhov, Chiara Sauli, Thierry Schmitt, Alessandro Silvano, Jodie Smith, Helen Snaith, Alex J. Tate, Kirsty Tinto, Philippe Vandenbossche, Pauline Weatherall, Paul Wintersteller, Chunguo Yang, Tao Zhang, Jan Erik Arndt

**Affiliations:** 1 Alfred-Wegener-Institut Helmholtz-Zentrum für Polar- und Meeresforschung, Bremerhaven, Germany; 2 School of Environment, Faculty of Science, University of Auckland, Auckland, New Zealand; 3 National Institute of Oceanography and Applied Geophysics OGS, Sgonico, Trieste, Italy; 4 Department of Earth System Science, University of California Irvine, Irvine, CA USA; 5 College of Surveying and Geo-Informatics, Tongji University, Shanghai, China; 6 Servicio Hidrográfico y Oceanográfico de la Armada de Chile, Santiago de Chile, Chile; 7 Polar Marine Geosurvey Expedition, St.Petersburg, Russian Federation; 8 GNS Science, Lower Hutt, New Zealand; 9 Geological Survey of Spain (CN IGME-CSIC), Madrid, Spain; 10 National Collections and Marine Infrastructure, CSIRO, Hobart, Tasmania Australia; 11 Instituto Andaluz de Ciencias de la Tierra (CSIC-UGR), Granada, Spain; 12 British Antarctic Survey, Cambridge, United Kingdom; 13 University of Plymouth, School of Biological and Marine Sciences, Plymouth, United Kingdom; 14 Key Lab of Submarine Geosciences, Second Institute of Oceanography, MNR, Hangzhou, Zhejiang China; 15 Istituto di Scienze Marine (CNR ISMAR), Bologna, Italy; 16 Scripps Institution of Oceanography, University of California, San Diego, La Jolla USA; 17 IHO Data Centre for Digital Bathymetry, NOAA’s National Centers for Environmental Information, Boulder, Colorado USA; 18 Korea Polar Research Institute, Incheon, Korea; 19 Department of Geological Sciences, Stockholm University, Stockholm, Sweden; 20 HafenCity University Hamburg, Hamburg, Germany; 21 Australian Antarctic Division, Kingston, Tasmania Australia; 22 All Russian Research Institute for Geology and Mineral Resources of the World Ocean, St.Petersburg, Russian Federation; 23 IFREMER, Centre Bretagne, Plouzané, France; 24 National Institute of Water & Atmospheric Research Ltd (NIWA), Wellington, New Zealand; 25 Center for Coastal and Ocean Mapping, University of New Hampshire, Durham, NH USA; 26 Institut des Géosciences de l’Environnement, Grenoble, France; 27 Department of Earth Sciences, Dartmouth College, Hanover, NH USA; 28 Lamont-Doherty Earth Observatory of Columbia University, Palisades, NY USA; 29 National Institute of Polar Research, Tokyo, Japan; 30 Geoscience Australia, Canberra, Australia; 31 Australian Antarctic Program Partnership, Institute for Marine and Antarctic Studies, University of Tasmania, Hobart, Tasmania Australia; 32 California Institute of Technology’s Jet Propulsion Laboratory, Pasadena, CA USA; 33 Department of Civil and Environmental Engineering, University of California Irvine, Irvine, CA USA; 34 Arctic and Antarctic Research Institute, St.Petersburg, Russian Federation; 35 Service Hydrographique et Océanographique de la Marine, Brest, Bretagne France; 36 Ocean and Earth Science, University of Southampton, Southampton, United Kingdom; 37 British Oceanographic Data Centre, National Oceanography Centre, Southampton, United Kingdom; 38 British Oceanographic Data Centre, National Oceanography Centre, Liverpool, United Kingdom; 39 MARUM and Faculty of Geosciences, University of Bremen, Bremen, Germany; 40 Federal Institute of Hydrology, Koblenz, Germany

**Keywords:** Geomorphology, Cryospheric science

## Abstract

The Southern Ocean surrounding Antarctica is a region that is key to a range of climatic and oceanographic processes with worldwide effects, and is characterised by high biological productivity and biodiversity. Since 2013, the International Bathymetric Chart of the Southern Ocean (IBCSO) has represented the most comprehensive compilation of bathymetry for the Southern Ocean south of 60°S. Recently, the IBCSO Project has combined its efforts with the Nippon Foundation – GEBCO Seabed 2030 Project supporting the goal of mapping the world’s oceans by 2030. New datasets initiated a second version of IBCSO (IBCSO v2). This version extends to 50°S (covering approximately 2.4 times the area of seafloor of the previous version) including the gateways of the Antarctic Circumpolar Current and the Antarctic circumpolar frontal systems. Due to increased (multibeam) data coverage, IBCSO v2 significantly improves the overall representation of the Southern Ocean seafloor and resolves many submarine landforms in more detail. This makes IBCSO v2 the most authoritative seafloor map of the area south of 50°S.

## Abstract (summary)

**Table:** 

Measurement(s)	depth of water
Technology Type(s)	echosounder
Factor Type(s)	bathymetry
Sample Characteristic - Environment	sea floor
Sample Characteristic - Location	Southern Ocean

## Background & Summary

The Southern Ocean is a major component of the coupled ocean-atmosphere climate system^1^ and includes the largest ocean current on earth, the Antarctic Circumpolar Current (ACC). It is furthermore the most important ocean region for the uptake of anthropogenic CO_2_ and heat from the atmosphere^2,3^, and cold and dense bottom waters form on the shelves surrounding Antarctica^4,5^. Interactions of the Southern Ocean with Antarctic glaciers and ice shelves are the main drivers of present, past, and future Antarctic ice sheet mass balance^6^ and thus global sea-level change. Biologically, the Southern Ocean is a high-productivity area^7^ with high biodiversity^8^. The Southern Ocean is also one of the most remote and harshest areas of the world with extensive sea-ice cover and year-round severe weather conditions. Despite its remoteness and hostility, human activities are increasingly extending into this distant part of the world, examples including research, fisheries, and tourism. Precise bathymetric information as e.g. provided by the International Bathymetric Chart of the Southern Ocean (IBCSO) and the Digital Bathymetric Model of the Drake Passage (DBM-BATDRAKE)^9^ are paramount to better understand the Southern Ocean and its processes as well as for human activities and conservation and management measures^10^. IBCSO aims to provide the most comprehensive compilation of bathymetric data for this region.

IBCSO was initiated in 2006 with the first version published by Arndt *et al*. in 2013^11^. It is the southern equivalent of the International Bathymetric Chart of the Arctic Ocean (IBCAO), which was originally produced in 2000 and recently released its fourth version^12,13^. Both initiatives are regional mapping projects of the General Bathymetric Chart of the Oceans (GEBCO). GEBCO is a project under the auspices of the International Hydrographic Organization (IHO) and the Intergovernmental Oceanographic Commission (IOC) with the goal to produce the authoritative map of the world’s oceans. Furthermore, IBCSO has combined its efforts with and is supported by the Nippon Foundation – GEBCO Seabed 2030 Project launched in 2017 by the Nippon Foundation of Japan and GEBCO^14^. The IBCSO Project is also an integral part of the Antarctic research community and an expert group of the Scientific Committee on Antarctic Research (SCAR).

Initially, IBCSO was limited to the Antarctic Treaty area covering the area south of 60°S with a resolution of 500 m × 500 m in a Polar Stereographic projection^11^. Following the release of Version 1, the user community expressed the wish for an IBCSO reaching to 50°S to cover the entire ACC and the Antarctic circumpolar frontal systems. This request, the growing demand for bathymetric information of the Southern Ocean, and the availability of numerous new bathymetric datasets collected since the first version of IBCSO were the motivations to produce a new version of IBCSO.

**Fig. 1:**
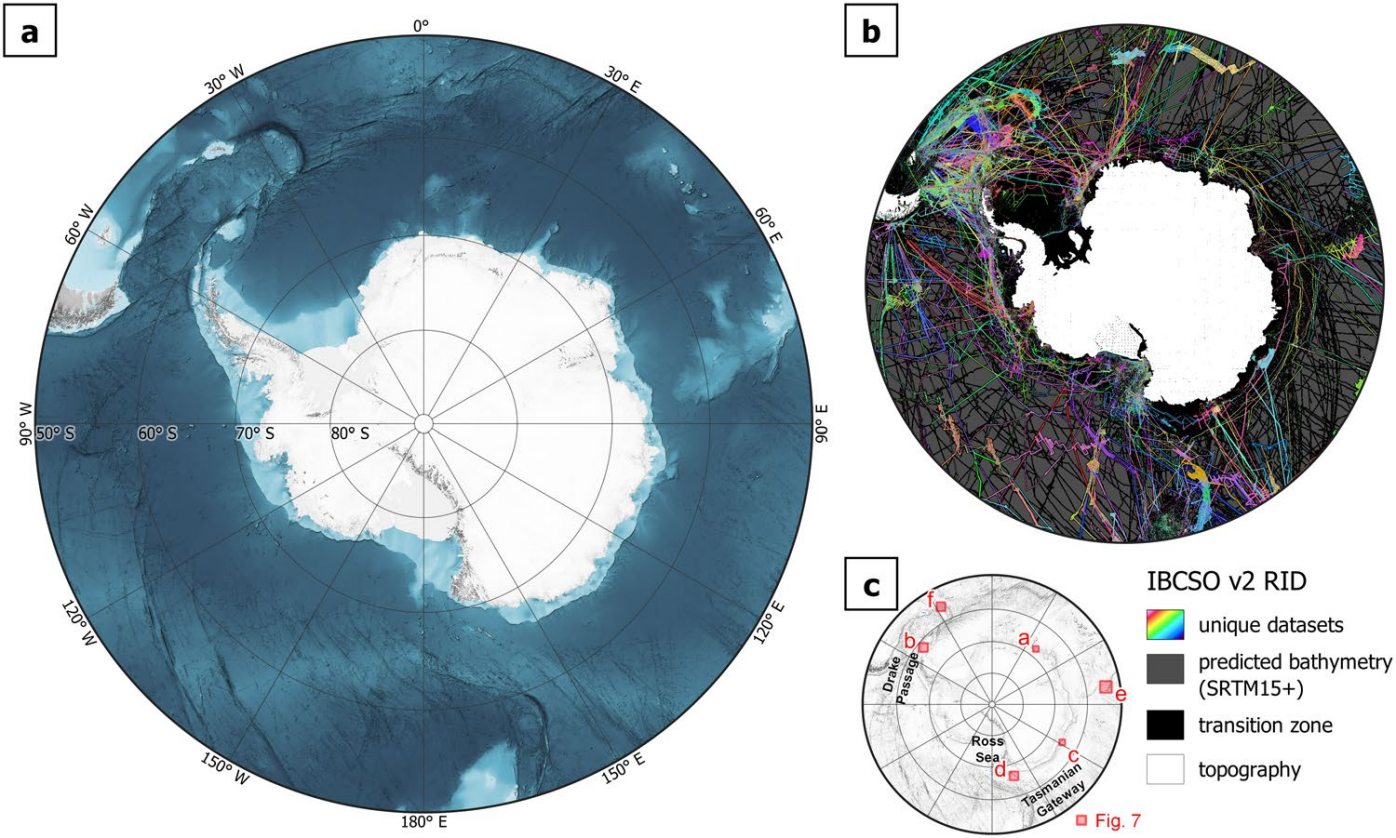
(**a**) Shaded relief of IBCSO v2 with ice surface topography. (**b**) Regional Identifier (RID) grid showing unique datasets (multicolours), topographic data (white), interpolated transition zone (black) and predicted bathymetry (dark grey). (**c**) Locations of example areas shown in Fig. 7.

Here we present IBCSO Version 2 (IBCSO v2) (Fig. 1) covering the area south of 50°S. The resolution is 500 m × 500 m in IBCSO Polar Stereographic projection (EPSG: 9354, see also the usage notes). It covers over 77 million km² of seafloor (approximately 2.4 times the area of seafloor covered by IBCSO v1). Highlights include improved bathymetries for the important oceanographic gateways of the ACC, the Drake Passage (now entirely included in IBCSO v2), and the Tasmanian Gateway (Fig. 1). The IBCSO v2 Digital Bathymetric Model (DBM) is available in two topography versions: one with ice surface elevation on the Antarctic continent and one with bedrock elevation, including sub-ice topography^15^. Furthermore, we provide a Type Identifier (TID) grid that indicates the type of data that composes each grid cell. The TID codes adhere to GEBCO standards (Table 1). In addition, a unique Regional Identifier (RID) grid links each data cell to the corresponding metadata information and thus the DBM’s cell value origin. All grids, a metadata table, and a digital chart of IBCSO v2 are publicly available for download from the PANGAEA data repository^16^.

**Table 1:** Type identifier (TID) table with codes adhering to the standards of the General Bathymetric Chart of the Oceans (GEBCO), short data type name, description, weight (see also Table 2), and number of linked datasets featured in IBCSO v2.

TID	data type	description	weight	no. of datasets
10	Singlebeam	Depth value collected by a single beam echo-sounder	10	766
11	Multibeam	Depth value collected by a multibeam echo-sounder	5, 15, 20, 25, 30	464
12	Seismic	Depth value collected by seismic methods	10	21
13	Isolated sounding	Depth value that is not part of a regular survey or trackline	5	2
14	ENC sounding	Depth value extracted from an Electronic Navigation Chart (ENC)	5	3
17	Combination of direct measurements	Combination of direct measurement methods	5	2
40	Predicted bathymetry based on satellite-derived gravity data	Depth value is an interpolated value guided by satellite-derived gravity data	no weight	[1]
41	Interpolated based on a computer algorithm	Interpolated based on a computer algorithm - depth value is an interpolated value based on a computer algorithm (e.g. Generic Mapping Tools)	no weight	[1]
42	Digital bathymetric contours from charts	Depth value taken from a bathymetric contour dataset	5	1
45	Predicted bathymetry based on flight-derived gravity data	Depth value is an interpolated value guided by helicopter/flight-derived gravity data	10, 5	3
46	Draft of a grounded iceberg	Depth estimated by calculating the draft of a grounded iceberg using satellite-derived freebord measurement	10	1
70	Pre-generated grid	Depth value is taken from a pre-generated grid that is based on mixed source data types, e.g. single beam, multibeam, interpolation etc.	5	[2]
71	Unknown source	Depth value from an unknown source	5	1
72	Steering points	Steering points - depth value used to constrain the grid in areas of poor data coverage	no weight	[2]

## Methods

**Fig. 2:**
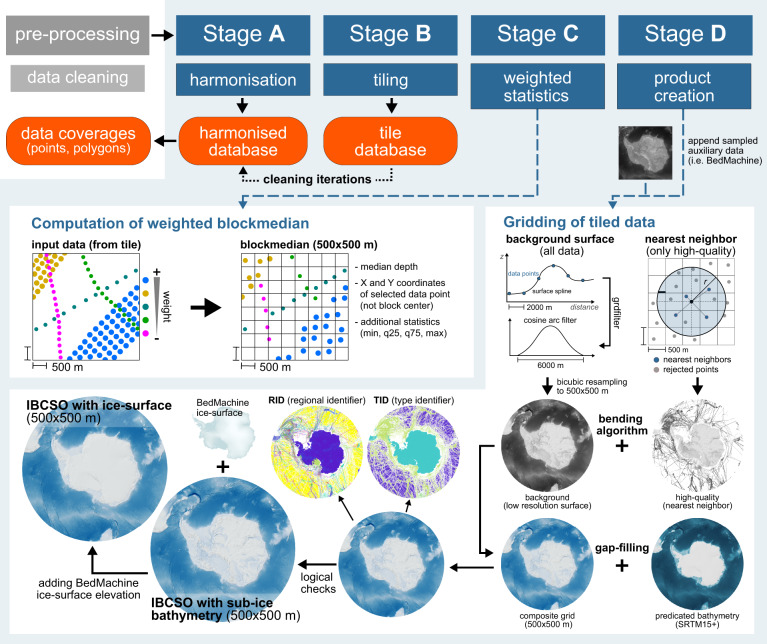
Schematic overview of the SEAHORSE processing workflow comprising the Stages A–D. Ice surface elevation and sub-ice bathymetry from BedMachine^15^, gap-filling with SRTM15+^23^.

The increase in coverage from IBCSO v1 to IBCSO v2 resulted in a substantial increase in the amount of data processing necessary at all levels from data submission to product generation. To cope with this higher computing workload, we have created a full computational environment surrounding the main processing pipeline (SEAHORSE, Fig. 2) of the IBCSO v2 DBM. This environment includes a database management system linked to SEAHORSE. To reduce run times, SEAHORSE is running dedicated code in a high-performance computing environment using parallel computing.

On submission, the quality and integrity of datasets is assessed visually and autonomously using designated Python scripts in order to identify major errors (e.g. inverted coordinates, wrong projections, outliers). After these initial checks, weights (Table 2) are assigned to datasets for later processing. Weights are based on the type of data (Table 1) as well as the quality and age of the data^16^. Multibeam datasets have generally high weights (≥15) compared to e.g. singlebeam data (weights ≤10) in order to supersede during data processing. Then, the data are transferred as ASCII XYZ files to SEAHORSE for the production of the IBCSO DBM.

**Table 2:** Numerical weights assigned to each source dataset based on data type, age, and quality.

weight	description
5	Exceptions for known bad quality data
10	Singlebeam
15	Multibeam older than 1993
20	Multibeam from 1993 to 2000
25	Multibeam younger than 2000
30	Exceptions for known good quality data
35	Above 0 (should replace any erroneous bathymetry)

### SEAHORSE processing workflow

SEAHORSE consists of four distinct stages (Stage A-D, Fig. 2), each containing a number of individual steps. All stages can be run independent from each other. Outputs include extensive reports for quality assurance (QA) and continuous feedback to the IBCSO metadata database (i.e. properties of the data sets derived from processing). SEAHORSE harmonises submitted datasets (harmonisation – Stage A), subdivides them into smaller spatial chunks of data (tiling – Stage B), calculates weighted blockmedians within these chunks (weighted statistics – Stage C) and computes a composite of all data (containing data of all quality). Furthermore, a subset that contains only high-quality data (weights ≥ 15) is computed for subsequent gap-filling to produce the final grid product (product creation – Stage D).

#### Stage A: harmonisation

The initial Stage A (Fig. 2) harmonises incoming datasets line by line and adds the harmonised version of the input data to the IBCSO file database. The harmonisation arranges arbitrarily ordered datasets into standardised XYZ files (consisting of an X, Y, and Z column) with each line representing a single geographic location and depth sounding. A next step identifies and removes potential errors in the data, such as erroneous depths (values exceeding known maximum depths in the study area) and implausible coordinate values (e.g. ship-borne bathymetry with locations on land). Output files from Stage A contain X, Y and Z values rounded to 1-metre accuracy, with duplicates removed, and separated by a standardised column separator. They are stored in a harmonised file database using the dataset identifier and the associated weight as filenames (Table 2).

#### Stage B: tiling

Stage B (Fig. 2) subdivides the harmonised file database into smaller spatial regions, pooling data from different sources. For this purpose, we subdivide the area south of 50°S into 100 km × 100 km tiles (in EPSG:9354 projection). Subsequently, a spatial join of all datasets with the defined tiles allows the assignment of each data point to a distinct tile. Points that do not fall into any tile are skipped and reported to QA. The result is a tile database with a single file for each tile. The tiles are further used to identify the origin of outliers and erroneous data visible in the final product. Erroneous data are removed from the harmonised database during iterative cleaning routines using the software suite Qimera® until all obvious artefacts disappear and a satisfactory quality is achieved.

#### Stage C: weighted statistics

In Stage C (Fig. 2), a weighted blockmedian is calculated for each 500 m × 500 m cell using the Generic Mapping Tools 6.1.1 (GMT) *blockmedian* module^17^. Five statistic descriptors are calculated: minimum, 25% quartile, 50% quartile (median), 75% quartile, and maximum of the weighted data in each cell. In a subsequent step, the median data points are augmented with additional information from the metadata, i.e. TID and RID. The outputs of this stage are single files per tile containing XYZ values, the summary statistics (min, q25, q75, and max), and categorical values (TID, RID, and the contributing organisation) for each line.

#### Stage D: product creation

In the final Stage D (Fig. 2), all files from Stage C are combined and subsets of geographic points are created (XYZ files) depending on the type of data. Based on the TID, the data are filtered to extract only high-quality data (weights ≥15, Table 2) from the database. The complete dataset and the extracted high-quality dataset are gridded using a modified processing sequence that has been initially introduced for the IBCAO Project by Jakobsson *et al*.^12^ and later adapted for IBCSO v1^11^, and the Southwest Indian Ocean Bathymetric Compilation^18^. For IBCSO v2, this approach has been further developed. At first, all irregularly spaced geographic points are gridded using a “*continuous curvature spline in tension”* from the GMT’s *surface* module^17^ with a tension factor of 0.35 (first used in IBCSO v1) to create a 2 km × 2 km background grid. Comparisons of outputs show that this tension factor is appropriate for the SEAHORSE workflow. This grid is subsequently filtered in the spatial domain using GMT *grdfilter* with an isotropic cosine arch convolution filter (6000 m width). The output is resampled to 500 m × 500 m resolution using a bicubic interpolation (GMT *grdsample*). The high-quality data are gridded to a separate 500 m × 500 m resolution grid using GMT *nearneighbor* to preserve the high-quality direct measurements in the final product.

**Fig. 3:**
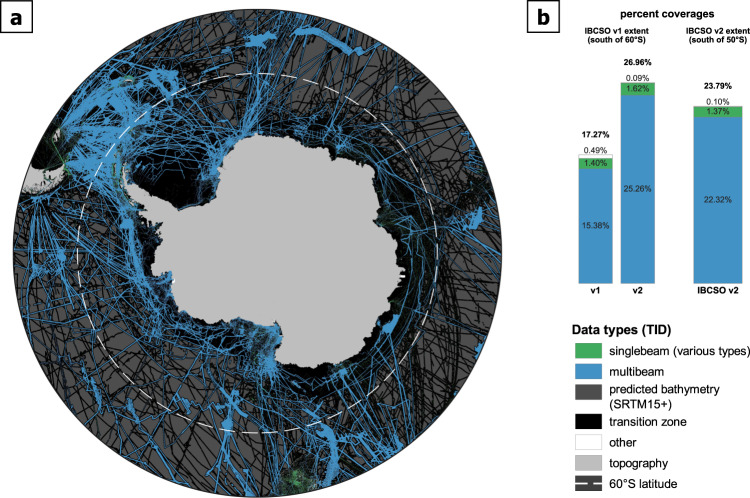
(**a**) Map showing the data type identifier (TID) of source data used for IBCSO v2. Various data types representing isolated soundings (TID: 10, 12, 13, 14) are grouped together and displayed as “singlebeam”. Data type “other” includes all TID greater than 14 (e.g. 71: unknown source) whereas “multibeam” only represents actual multibeam datasets (TID: 11). White dashed line represents the northernmost IBCSO v1 extent (60°S latitude). (**b**) Comparison of percent seafloor coverages by different data types for IBCSO v1 and v2 south of 60°S as well as current status of IBCSO v2 (south of 50°S).

Background and high-quality grids are combined using the bending algorithm from Arndt *et al*.^11^ that follows the remove-restore concept described in Hell and Jakobsson^19^ and Jakobsson *et al*.^12^. The algorithm is implemented using the programming language Python and its scientific ecosystem, e.g. SciPy^20^, NumPy^21^, PyGMT^22^, and Dask (https://dask.org/) as an interface for GMT^17^. Based on experiences from previous compilations, we choose a transition zone covering 20% high-quality and 80% background data grid along the intersection edges for the bending (Fig. 3). An extended high-quality grid is calculated by convolving both grids to infill (i.e. extrapolate) the transition zone for the sparse high-quality grid. This is required to calculate the depth values (*z*_*c*_) in Eq. (1) for the transition zone using a combination of the extended high-quality and background grids where *z*_*h*_ and *z*_*b*_ are depth values of the high-quality and the background grid, respectively,

1 $${z}_{c}=\frac{{z}_{b}\ast {d}_{i}^{2}+{z}_{h}\ast {d}_{o}^{2}}{{d}_{i}^{2}+{d}_{o}^{2}}$$

with weighted distances of the transition zone grid cells to the inner (*d*_*i*_) and outer (*d*_*o*_) edges of the transition zones, using a hyperbolic weighting function (1/d^2^). Depth values in the transition zone are progressively more affected by the closer (e.g. high-quality) input grid. Finally, the high-quality and background grids are merged by replacing cell values in the background grid with values from the high-quality grid before inserting the transition zone cell values calculated via Eq. (1). This approach successfully minimises undesired edge effects caused by the combination of grids of different resolutions and potential depth offsets. The quality of the resulting composite grid is visually evaluated using the Open Source Geographic Information System QGIS.

In the following gap-filling step, areas without direct measurements are filled with predicted bathymetry (for IBCSO v2 this is SRTM15+ v2.2^23^). The composite and predicted bathymetry grids are combined using the above-described bending algorithm with a transition zone of 10 km (or 20 grid cells for 500 m resolution) that exclusively comprises grid cells from the predicted bathymetry grid to avoid altering high-quality data cells representing direct depth measurements. Pre-bending, the predicted bathymetry grid is adjusted to the IBCSO database to minimise artefacts caused by varying depths by calculating an offset factor between both grids on a cell-by-cell basis. A 1000 m × 1000 m blockmedian is computed with the GMT *blockmedian* module to suppress small-scale artefacts in the grid. The factor values are re-gridded and filtered using GMT *surface* and *grdfilter* with a cosine arch filter (2000 m × 2000 m) via PyGMT before the resulting grid is resampled to 500 m × 500 m using GMT *grdsample*. Then, this factor grid is used to adjust the predicted bathymetry grid by multiplying both grids. Areas are masked out, if the adjusted predicted bathymetry differs significantly from the surface grid (e.g. continental shelf areas and around islands). There, the background surface spline grid is used instead. This approach successfully prevents artefacts caused by differences in data resolution and accuracy.

In the final step, ice-surface and ocean mask grids are dynamically generated from the datasets created in previous processing steps (Fig. 2). The ice-surface mask is derived from the BedMachine^15^ surface elevation grid. The ocean mask is calculated from the gap-filled composite grid considering the ice-surface mask and RID grid (excluding all values above 0 m). It is used to assure that all ocean cells are modelled below sea level and all topographic cells are modelled above sea level. Grid cells that failed this logical test are set to the value −1 for ocean cells and to the value 1 for topographic cells. The ice-surface mask is used to create IBCSO v2 with ice surface elevation from BedMachine^15^.

## Data Records

IBCSO v2 is available for download from the PANGAEA data repository^16^. It comprises a variety of datasets (Table 1) ranging from digitised contours and lead line soundings to high-resolution multibeam data. If possible, the use of gridded compilations was avoided and source datasets were used instead to achieve the most consistent interpolation and prevent an overestimation of the covered area (Fig. 3). Therefore, each dataset mostly refers to a single expedition with its unique RID value^16^.

### Bathymetry

High-resolution multibeam datasets make up the basis of the compilation with a total of 464 datasets. In addition, 766 singlebeam datasets provide measured bathymetric information (Table 1, Fig. 3). The datasets were received in various formats and were standardised as ASCII XYZ data with associated metadata information when available (e.g. data contributor, source survey, year of survey). However, many datasets lack detailed information regarding their origins making it difficult to assess their quality. Furthermore, the spatial distribution of data shows a high degree of heterogeneity. For example, Drake Passage and the Ross Sea areas display high multibeam data coverage while along East Antarctica mostly singlebeam data exist (Fig. 3).

IBCSO v2 uses SRTM15+ v2.2^23^ as the predicted bathymetry. It, however, contains numerous artefacts especially in areas of sea-ice cover and on the continental shelves. To avoid the incorporation of those artefacts, after interrogation of the available high-resolution multibeam data, critical areas are masked out for the infill with predicted bathymetry.

### Sub-ice shelf bathymetry

Sub-ice shelf bathymetry in IBCSO v2 is constrained by direct measurements (e.g. from seismic campaigns), and in the absence of direct measurements by bathymetry estimations from gravity inversions, interpolation, and artificial steering lines. Seismic measurements from 21 datasets conducted since the 1950s are included (Supplementary Table 1).

We only include bathymetry inferred from gravity inversion that rely on airborne gravity measurements and only in areas that are further away than 5 km from direct measurements in the IBCSO v2 database. In addition, we do not use bathymetry inferred from gravity inversions in areas where the models produce unrealistically shallow topography. Such areas have been identified either by a large discrepancy between the depths modelled by the gravity inversion and depths determined by seismic measurement, or by very small water column thicknesses (less than 100 m) in the sub-ice shelf continuation of narrow, deeply incised subglacial troughs beyond the grounding-line. Such areas with steep topography and abrupt elevation changes are usually poorly resolved by gravity inversions due to the long wavelength and are therefore typically inadequately modelled^24,25^. This mainly occurs at the western Ross Ice Shelf close to the Transantarctic Mountains. Supplementary Table 2 summarises the gravity inversions that are incorporated directly or as part of the BedMachine Antarctica dataset^15^.

For the Amery Ice Shelf cavity, we use the bathymetric model created by Galton-Fenzi *et al*.^26^. This model also uses seismic point data and an interpolation guided by tidal modelling for the deepest, most inland section of the ice shelf which is difficult to survey due to crevasses. For the remaining ice shelf areas, i.e. where neither direct measurements nor good quality gravity inversions exist, we have investigated the adjacent bathymetry and subglacial bedrock measurements for glacially incised troughs. Where such troughs are located, we introduce artificial steering lines to guide our interpolation to model a continuation of these troughs. For the remaining areas, we use the seafloor depths as provided in the bed layer of BedMachine^15^.

### Sub-ice sheet topography, ice surface topography, and island topography

Sub-ice sheet topography is entirely derived from the bed layer of BedMachine^15^. BedMachine in these areas builds on ice-thickness measurements from airborne radio-echo sounding and a mass-conservation approach that uses ice sheet dynamics to interpolate between measurements^15^.

The topography is derived from various datasets (Fig. 3). Their selection depends on the geographical region and the quality of the different datasets in these regions. For the Antarctic mainland, we use the surface layer of the BedMachine dataset^15^ derived from the “Reference Elevation Model of Antarctica“ (REMA) that has a spatial resolution of 8 m^27^. Despite this higher resolution, we use the BedMachine topography information in continental ice-covered areas of Antarctica to ensure consistency with the ice thicknesses reported within BedMachine. For some coastal, ice-free areas of East Antarctica and for some small islands that are not resolved in BedMachine, we have directly added elevation information from the REMA dataset^27^.

For many Antarctic islands, REMA, and thus also BedMachine, yields no (e.g. South Orkney Islands, Balleny Islands) or incomplete (e.g. King George Island) topographic information. For these islands, most sub-Antarctic islands, and for South America we use elevation data from the ALOS Global Digital Surface Model Version 3.2 of the Japan Aerospace Exploration Agency^28^. For a few islands, including some smaller reefs, and for a few parts of larger islands, the ALOS model does not provide elevation information. These areas are modelled using elevation data from other models, for example reefs at South Georgia from a compilation by Fretwell *et al*.^29^ and parts of the South Sandwich Islands from a compilation by Leat *et al*.^30^. In the cases where no elevation models are available but the location of the island is constrained by satellite imagery, we create artificial elevation data to constrain our model to a reasonable elevation.

## Technical Validation

SEAHORSE produces detailed reports for each individual stage. These reports are used to get estimates on runtimes per step and the size of data processed in each stage. In addition, we create a wide range of auxiliary data used for internal quality management and data review.

For estimating the variation of data from different surveys within a grid cell, we use the interquartile range (the absolute distance between the 25% and 75% quartile) of blockmedian window data to produce bathymetric charts analogous to the main workflow. These we use to derive a map of depth-ranges (*z*_*range*_) in Eq. (2).

2 $${z}_{range}=\left|({Q}_{75}-{Q}_{25})\right|$$

**Fig. 4:**
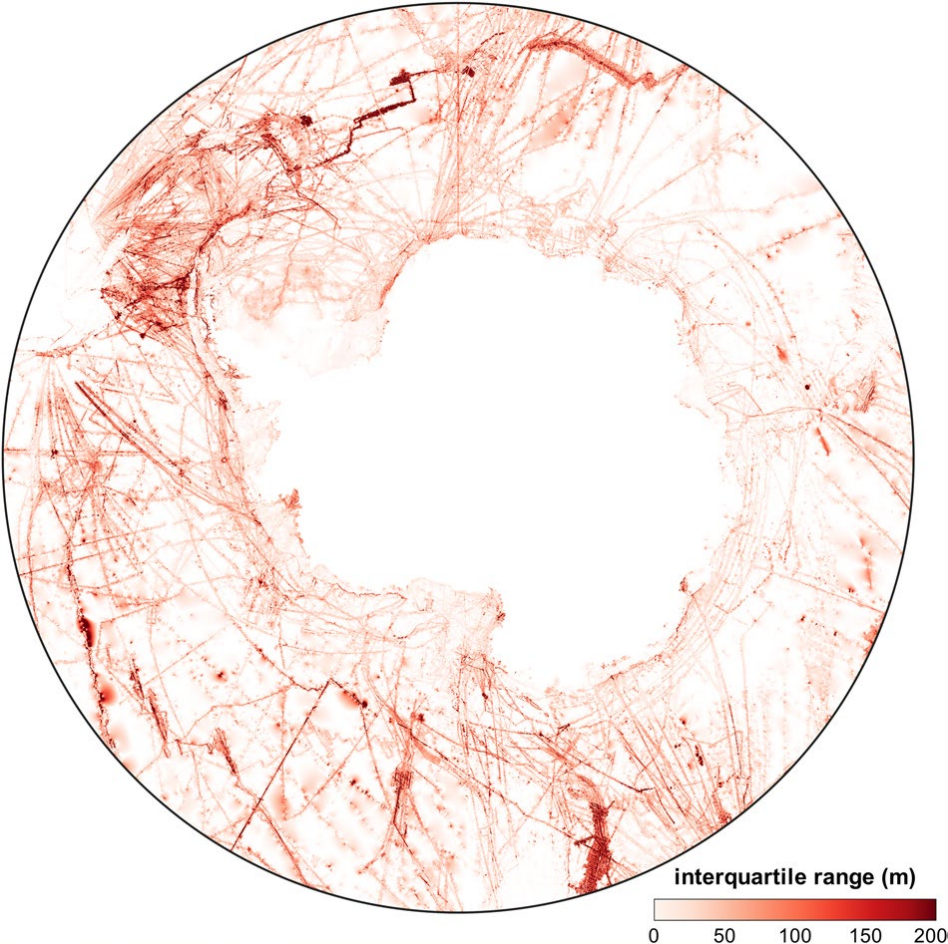
Map showing the interquartile range for the final depth values of the grid. Estimation based on grids created from the 25% quartile and the 75% quartile of data as reported by GMT blockmedian.

Under the assumption that depth values per grid cell are normal distributed with zero skewness, this is the most intuitive measure of variability that we can derive. While this is not a perfect way to measure the uncertainty in a given grid cell, we regard this as the most practical way to get an estimate of the expected range of depth values for every given grid cell (Fig. 4).

Overall variability increases with the number of datasets in a grid cell. The interquartile range of depth values at any given cell falls mostly between 0 m and 100 m. High values occur along the regular supply routes for Antarctic stations and within areas of high scientific interest where many datasets overlap. These areas have been visited across multiple generations of technical proficiency. On the other hand, areas with low variability indicate areas with little survey effort or areas that have produced similar data across multiple data sources. This can be expected for measurements e.g. in shallower waters. However, variability does not immediately quantify the reliability of the reported depth value. We can conclude that our blockmedian approach is robust against outliers in the 25^th^ and 75^th^ quartiles. Only areas where both low coverage and high variation in measured data coincide have a detrimental effect on the final depth value in a grid cell.

**Fig. 5:**
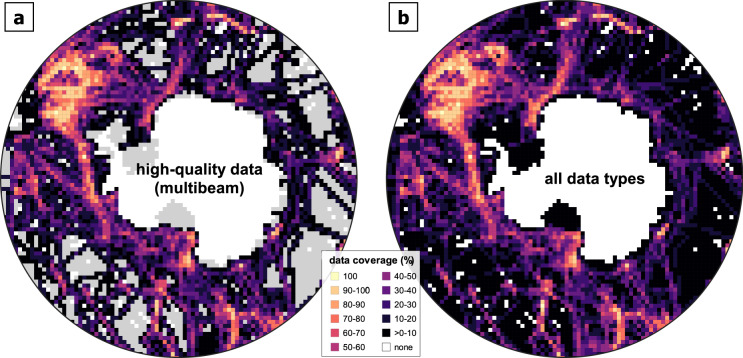
Overall data coverage of IBCSO v2 indicated by coverage per tile (100 km × 100 km). (**a**) Data coverage of only high-quality multibeam datasets (weights ≥15, multibeam data, see Tables 1 and 2) with tiles featuring only low-quality data (weights <15) masked out in grey. (**b**) Data coverage based on all datasets.

The RID grid (Fig. 1b) gives a first impression, where the IBCSO v2 grid is constrained by actual data. Data coverage per tile (Fig. 5) provides an additional indication of how many grid cell values per tile originated from measured data. This coverage map highlights distinct distribution patterns. Exceptionally high coverages, up to 100%, occur e.g. in the Drake Passage (upper left sector Fig. 5), whereas vast areas with sparse data coverage are especially prominent offshore East Antarctica. Depths in regions of high data coverage can be considered reliable, regardless of an apparent increase in the interquartile range. These areas are located along more frequently used ship routes and have been surveyed using more accurate recent multibeam systems. In areas with low data coverages (Fig. 5), the inclusion of the SRTM15 + predicted bathymetry grid yields a more comprehensive and representative DBM of the seafloor.

**Fig. 6:**
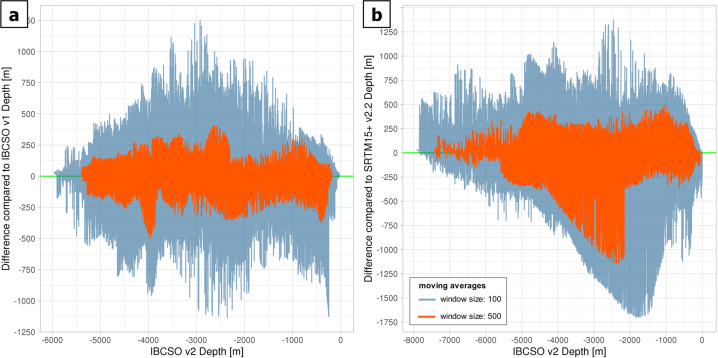
Cell by cell difference between IBCSO v2 depths (x-axis) and reference grid depth differences (on y-axis). (**a**) IBCSO v1 as reference grid; (**b**) SRTM 15+ as reference grid. Blue lines indicate moving average with step size 100, orange lines indicate moving averages with step size 500. Grids were masked to contain only ocean cells and extents were adjusted in order to ascertain identical extents when comparing IBCSO v2 and the reference grid.

The overall increase in multibeam data coverage (Fig. 3b) resulted in a clear improvement of the grid. The differences between IBCSO v2 and the reference grids IBCSO v1 (Table 3) and SRTM 15+ were assessed to quantify the impact of the new data contributions (and updates of external data, such as the predicted bathymetry grid and high-resolution topographic data). When comparing grids, we applied the ocean mask from Stage D to use the same extent. Then, the arithmetic difference between each cell of IBCSO v2 and its corresponding grid cell from the reference grid (discarding all empty cell pairs) were calculated. Due to the amount of data, moving averages (with window sizes of 100 and 500) were plotted of the depth difference for each grid cell (Fig. 6a,b). The plot is created with ggplot2^31^ in R version 3.6.1 (https://www.r-project.org/). Difference between IBCSO v2 and IBCSO v1 (Fig. 6a) are noticeable throughout all depth ranges. The comparison with SRTM15+ (Fig. 6b) also shows noticeable differences for water depths in particular between −4500 m and −2000 m.

**Table 3:** Descriptive summary of metadata and the database of IBCSO v2.

Item	Value
Number of contributors	39
Number of sources	>90
Number of contributing countries	18
Seafloor area covered by IBCSO v1	32,676,775 km^2^
Seafloor area covered by IBCSO v2	77,321,752 km^2^
IBCSO v2 data coverage	23.79%
Multibeam coverage	22.32%
Singlebeam coverage	1.37%
Other data type coverage	0.10%

Seafloor area is calculated based on WGS84 ellipsoid using the QGIS plugin Cruise Tools (https://github.com/simondreutter/cruisetools). Data type coverages correspond to percentage of filled ocean cells in IBCSO v2 grid resolution (500 m × 500 m).

**Fig. 7:**
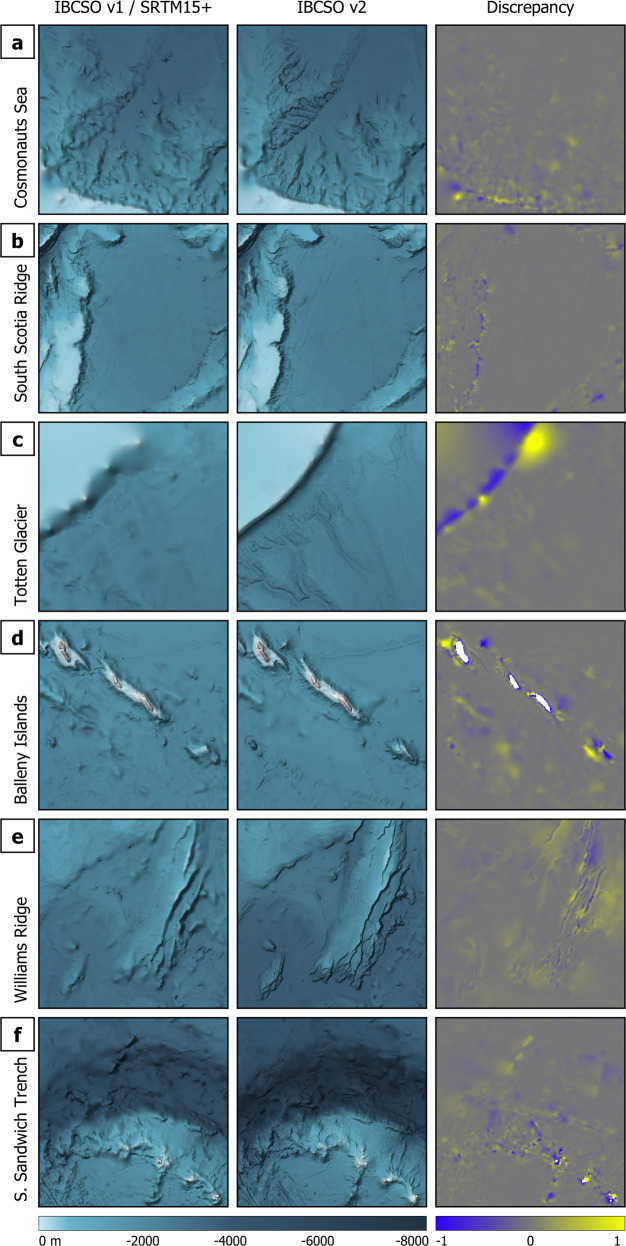
Comparison between IBCSO v1 and IBCSO v2 for: (**a**) Cosmonauts Sea, (**b**) South Scotia Ridge, (**c**) seaward of Totten Glacier and (**d**) Balleny Islands. Plots indicate (from left to right) IBCSO v1 chart, IBCSO v2 chart and calculated discrepancy between IBCSO v1 and IBCSO v2. Comparison between SRTM15+ and IBCSO v2 for: (**e**) Williams Ridge (Kerguelen Plateau) and (**f**) South Sandwich Trench and Islands. Plots indicate (from left to right) SRTM15+ chart, IBCSO v2 chart and calculated discrepancy between SRTM15+ and IBCSO v2. Grids for comparison are masked to contain only ocean cells. Columns IBCSO v1 and IBCSO v2 show the seabed as depth-scaled colour layer shaded by multiplication with a slope-inclination layer and a synthetic light source (hillshade) with 10× vertical exaggeration.

For more detailed comparisons between IBCSO v2 and the reference grids IBCSO v1 and SRTM15+, we target six areas of interest for closer inspection (Figs. 1c, 7). Since IBCSO v1 does not provide information on uncertainty, we cannot use any measure of uncertainty for this comparison. Instead, we opt for a discrepancy metric (discrepancy λ, Eq. (3)) defined as the difference grid between IBCSO v2 and IBCSO v1 or SRTM15+ (δ, Eq. (4)) divided by the mean of IBCSO v2 and IBCSO v1 or SRTM15+ (μ, Eq. (5)):

3 $$\lambda =\frac{\delta }{\mu }$$

4 $$\delta ={z}_{IBCSOv2}-{z}_{Reference}$$

5 $$\mu =\frac{({z}_{IBCSOv2}+{z}_{Reference})}{2}$$

This results in values centred on zero, with positive numbers indicating IBCSO v2 depths being deeper than the reference grid and negative numbers being shallower.

When comparing IBCSO v2 with v1, areas with significant bathymetric changes over a relatively short distance e.g. the shelf break around Antarctica (Fig. 7a,c), the South Scotia Ridge (Fig. 7b) or slopes around islands (Fig. 7d) display more pronounced discrepancies. The change in data coverage and quality is obvious when looking at the area seaward of Totten Glacier (Fig. 7c) where a multibeam dataset acquired in 2017 by the Australian research vessel RV Investigator improves the morphology of the shelf break and resolves a network of submarine channels at the slope. Similar improvement is visible at the South Scotia Ridge where incised slopes facing towards the Powell Basin have been mapped in high-resolution by the RV Polarstern^32^ in 2019 resulting in a larger discrepancy. The benefit of incorporating actual source data rather than gridded compilations is seen in Fig. 7d. In IBCSO v1, the slopes around the Balleny Islands are based on a data compilation with a resolution of 1000 m. However, for IBCSO v2 we were able to receive the source data in full multibeam resolution resulting in a much more detailed DBM for this area compared to IBCSO v1.

Improvements can also be observed when comparing IBCSO v2 with the predicted bathymetry grid (SRTM15 + v2.2, Fig. 7). The Williams Ridge and the adjacent Labuan Basin at the Kerguelen Plateau were mapped by the RV Investigator and the RV Sonne in 2020. These additional data substantially improved the bathymetry for this region (Fig. 7e). Distinct improvements are also visible when examining the region around the South Sandwich Islands and Trench (Fig. 7f). Although not covered by IBCSO v1, the comparison with the SRTM15 + grid highlights an increased grid quality caused by large seafloor areas now constrained by multibeam measurements. This effect is especially obvious at the slopes around the South Sandwich Islands. Overall, the IBCSO v2 grid contains a multiplicity of additional datasets gathered since the release of IBCSO v1 also including extended areas previously not covered.

## Usage Notes

The IBCSO DBM is provided in GeoTIFF and netCDF-4 file formats with coordinates and depth stored as 16-bit integers and a pixel node registration. These formats can be imported into all major GIS packages (e.g. QGIS, ArcGIS). All grids are available in geographic coordinates (WGS84, EPSG:4326) and in projected Cartesian coordinates defined in the IBCSO Polar Stereographic projection registered with the EPSG Geodetic Parameter Dataset using the code EPSG:9354 (https://epsg.org/crs_9354/WGS-84-IBCSO-Polar-Stereographic.html). The projection’s true scale is set at 65°S and coordinates in X and Y directions are given in meters. The horizontal datum is WGS84 whereas the vertical datum is approximately Mean Sea Level. Due to limited acquisition parameter information, there are uncertainties associated with the vertical datum information, especially for older data. The grid cell value of the DBM is given in meters with negative values representing depths below sea level and positive values corresponding to topographic elevation. For the RID and TID grids, the cell values represent a unique dataset and type identifier value, respectively. An overview of the TID codes is given in Table 1, whereas a list of all incorporated datasets is provided at the PANGAEA data repository^16^.

When using the native IBCSO projection, it is important to consider the following: The EPSG code was registered in March 2020 and first included in EPSG v9.8.11 database published on the 30^th^ April 2020 that was again included in the PROJ 7.1.0 database (from 1^st^ July 2020). However, QGIS versions prior to release 3.20.0 (from 19th June 2021) are using older PROJ database versions that do not include the IBCSO projection (EPSG:9354). Similar limitations may also apply to other GIS software packages (e.g. ArcGIS) depending on the version of their libraries. In this case, we recommend creating a temporary user-defined CRS from the specifications provided at the PANGAEA data repository^16^.

## Supplementary information

Supplmentary Tables 1 and 2

## Data Availability

The GMT and GDAL routines used in the SEAHORSE workflow are Open Source and can be accessed on their respective webpages (https://www.generic-mapping-tools.org/ and https://gdal.org/). All relevant code related to the main SEAHORSE workflow are available at https://github.com/SeaBed2030/IBCSO_v2_Dorschel_et_al_2022. Data for the technical validation are hosted on figshare^33^. Since the SEAHORSE workflow was customised to fit the existing architecture of AWI’s high performance cluster, most of the code is specific and requires severe adjustments when moved to a different environment.
