# Supplementary material for: The International Bathymetric Chart of the Southern Ocean Version 2
Source: Sci Data. 2022 Jun 7;9:275. doi: 10.1038/s41597-022-01366-7 (PMC9174482; doi:10.1038/s41597-022-01366-7)
Supplement: Supplementary file 1 — Supplmentary Tables 1 and 2 [file 41597_2022_1366_MOESM1_ESM.pdf]

Supplementary Information

Table of Contents

Supplementary Table 1: Ground-based seismic datasets used to constrain sub-ice shelf bathymetry....... 1

References for Supplementary Table 1....... 1

Supplementary Table 2: Bathymetric information from gravity inversions 4

References for Supplementary Table 2....... 4

Supplementary Table 1: Ground-based seismic datasets used to constrain sub-ice shelf bathymetry

Region	RID	Year(s)	References
Amundsen Sea Embayment		2017	(Muto et al., 2013)
Berkner Island (near)	11307	1998-1999	None
Brunt Ice Shelf	11295	2004	(Hodgson et al., 2018)
Ekström Ice Shelf	11298	2010-2018	(Smith, 1997)
Ekström and Quar Ice Shelf	11300	1951-1952	(Glen and Glen, 1959)
Filchner Ice Shelf	11308	1957-1958	(Behrendt, 1962)
Fimbul Ice Shelf	11296	2000-2001	(Nøst, 2004)
George VI Sound	11293	1984-1985	(Constantino et al., 2020; Maslanyj, 1987)
Getz Ice Shelf (eastern)	11299	1959-1960	(Bentley and Chang, 1971)
Larsen C Ice Shelf	11261, 11262	2012-2016	(Brisbourne et al., 2020; Brisbourne et al., 2014; Holland et al., 2015) https://doi.org/10.5285/315740B1-A7B9-4CF0-9521-86F046E33E9A (Brisbourne et al., 2019) https://doi.org/10.5285/5D63777D-B375-4791-918F-9A5527093298 (Booth, 2019) https://doi.org/10.5285/FFF8AFEE-4978-495E-9210-120872983A8D (Kulessa and Bevan, 2019) https://doi.org/10.5285/147BAF64-B9AF-4A97-8091-26AEC0D3C0BB (Booth et al., 2019)
Lazarev Ice Shelf	11335		(Bokanenko and Avsyuk, 1963)
Pensacola Mountains (near)	11309	1965-1966	(Behrendt et al., 1974)
Ronne Ice Shelf	11310	1994-1995	(Johnson and Smith, 1997)
Ronne Ice Shelf (southern)	11311	1963-1964	None
Ronne-Filchner Ice Shelf	11306,<br>11312	2015-2017<br>1976-1986	(Rosier et al., 2018) https://doi.org/10.5285/dada63fb-c40a-4b13-97ba-c53860881d79<br>(Pozdeev and Kurinin, 1987)
Ross Ice Shelf	11297, 11301<br>11302	1973-1978<br>1957-1960	(Albert and Bentley, 1990; Robertson and Bentley, 1990)<br>(Crary et al., 1962)
Roosevelt Island (near)	11303	1962-1963	None
Rutford Ice Stream mouth	11304	1991-1993	(Smith, 1997; Smith and Doake, 1994)

References for Supplementary Table 1

Albert D.G., Bentley C.R., 1990. Seismic Studies on the Grid Eastern Half of the Ross Ice Shelf: RIGGS III and RIGGS IV, The Ross Ice Shelf: Glaciology and Geophysics, pp. 87-108, https://doi.org/10.1029/AR042p0087.

Behrendt J.C., 1962. Geophysical and glaciological studies in the Filchner Ice Shelf Area of Antarctica. Journal of Geophysical Research (1896-1977) 67, 221-234, https://doi.org/10.1029/JZ067i001p00221.

Behrendt J.C., Henderson J.R., Meister L., Rambo W.L., 1974. Geophysical investigations of the Pensacola Mountains and adjacent glacierized areas of Antarctica, Professional Paper, - ed, https://doi.org/10.3133/pp844.

Bentley C.R., Chang F.-K., 1971. Geophysical Exploration in Marie Byrd Land, Antarctica, Antarctic Snow and Ice Studies II, pp. 1-38, https://doi.org/10.1029/AR016p0001.

Bokanenko L.I., Avsyuk Y.N., 1963. Subglacial relief and thickness of the Lazarev Ice shelf. Bulletin of the Soviet Antarctic Expedition 44, 43-48

Brisbourne A.M., Kulessa B., Hudson T., Harrison L., Holland P.R., Luckman A.J., Bevan S., Ashmore D., Hubbard B., Pearce E., White J.W.C., Booth A., Nicholls K.W., Smith A.M., 2020. An updated seabed bathymetry beneath Larsen C Ice Shelf, Antarctic Peninsula. Earth Syst. Sci. Data 12, 887-896, https://doi.org/10.5194/essd-12-887-2020.

Brisbourne A.M., Smith A.M., King E.C., Nicholls K.W., Holland P.R., Makinson K., 2014. Seabed topography beneath Larsen C Ice Shelf from seismic soundings. The Cryosphere 8, 1-13, https://doi.org/10.5194/tc-8-1-2014.

Constantino R.R., Tinto K.J., Bell R.E., Porter D.F., Jordan T.A., 2020. Seafloor Depth of George VI Sound, Antarctic Peninsula, From Inversion of Aerogravity Data. Geophysical Research Letters 47, e2020GL088654, https://doi.org/10.1029/2020GL088654.

Crary A.P., Robinson E.S., Bennett H.F., Boyd Jr. W.W., 1962. Glaciological regime of the Ross Ice Shelf. Journal of Geophysical Research (1896-1977) 67, 2791-2807, https://doi.org/10.1029/JZ067i007p02791.

Glen J.W., Glen J.W.G., 1959. Glaciological Research by the Norwegian-British-Swedish Antarctic Expedition: Review. The Geographical Journal 125, 239-243, https://doi.org/10.2307/1790509.

Hodgson D.A., Hogan K.A., Smith J.M., Smith J.A., Hillenbrand C.-D., Graham A.G.C., Fretwell P.T., Allen C.S., Peck V., Arndt J.E., Dorschel B., Hübscher C., Smith A.M., Larter R.D., 2018. Deglaciation and future stability of the Coats Land ice margin, Antarctica. The Cryosphere 12, 2383-2399, https://doi.org/10.5194/tc-12-2383-2018.

Holland P.R., Brisbourne A., Corr H.F.J., McGrath D., Purdon K., Paden J., Fricker H.A., Paolo F.S., Fleming A.H., 2015. Oceanic and atmospheric forcing of Larsen C Ice-Shelf thinning. The Cryosphere 9, 1005-1024, https://doi.org/10.5194/tc-9-1005-2015.

Johnson M.R., Smith A.M., 1997. Seabed topography under the southern and western Ronne Ice Shelf, derived from seismic surveys. Antarctic Science 9, 201-208, https://doi.org/10.1017/S0954102097000254.

Maslanyj M.P., 1987. Seismic bedrock depth measurements and the origin of George VI Sound, Antarctic Peninsula. British Antarctic Survey Bulletin 75, 51-65, http://nora.nerc.ac.uk/id/eprint/522872.

Muto A., Anandakrishnan S., Alley R.B., 2013. Subglacial bathymetry and sediment layer distribution beneath the Pine Island Glacier ice shelf, West Antarctica, modeled using aerogravity and autonomous underwater vehicle data. Annals of Glaciology 54, 27-32, https://doi.org/10.3189/2013AoG64A110.

Nøst O.A., 2004. Measurements of ice thickness and seabed topography under the Fimbul Ice Shelf, Dronning Maud Land, Antarctica. Journal of Geophysical Research: Oceans 109, https://doi.org/10.1029/2004JC002277.

Pozdeev V.S., Kurinin R.G., 1987. New data on ice sheet morphology, bedrock and bottom relief in the southern Weddell Sea Basin, West Antarctica. Antarktika. doklady komissii 26, 66-71

Robertson J.D., Bentley C.R., 1990. Seismic Studies on the Grid Western Half of the Ross Ice Shelf: RIGGS I and RIGGS II, The Ross Ice Shelf: Glaciology and Geophysics, pp. 55-86, https://doi.org/10.1029/AR042p0055.

Rosier S.H.R., Hofstede C., Brisbourne A.M., Hattermann T., Nicholls K.W., Davis P.E.D., Anker P.G.D., Hillenbrand C.-D., Smith A.M., Corr H.F.J., 2018. A new bathymetry for the southeastern Filchner-Ronne Ice Shelf: implications for modern oceanographic processes and glacial history. Journal of Geophysical Research: Oceans, https://doi.org/10.1029/2018JC013982.

Smith A.M., 1997. Basal conditions on Rutford Ice Stream, West Antarctica, from seismic observations. Journal of Geophysical Research: Solid Earth 102, 543-552, https://doi.org/10.1029/96JB02933.

Smith A.M., Doake C.S.M., 1994. Sea-bed depths at the mouth of Rutford Ice Stream, Antarctica. Annals of Glaciology 20, 353-356, https://doi.org/10.3189/1994AoG20-1-353-356.

Supplementary Table 2: Bathymetric information from gravity inversions

Region	Import Method	Reference
Amundsen Sea ice shelves	via BedMachine	(Millan et al., 2017)
Brunt Ice Shelf	direct	(Hodgson et al., 2019)
George VI Sound	direct	(Constantino et al., 2020)
Getz Ice Shelf	via BedMachine	(Millan et al., 2020)
Ross Ice Shelf	via BedMachine	(Tinto et al., 2019)
Shackleton Ice Shelf	via BedMachine	(Brancato et al., 2020)
Sulzberger Ice Shelf	via BedMachine / Bedmap2	(Luyendyk et al., 2003)
Totten Glacier	via BedMachine	(Greenbaum et al., 2015)

References for Supplementary Table 2

Brancato V., Rignot E., Milillo P., Morlighem M., Mouginot J., An L., Scheuchl B., Jeong S., Rizzoli P., Bueso Bello J.L., Prats-Iraola P., 2020. Grounding Line Retreat of Denman Glacier, East Antarctica, Measured With COSMO-SkyMed Radar Interferometry Data. Geophysical Research Letters 47, e2019GL086291, https://doi.org/10.1029/2019GL086291.

Constantino R.R., Tinto K.J., Bell R.E., Porter D.F., Jordan T.A., 2020. Seafloor Depth of George VI Sound, Antarctic Peninsula, From Inversion of Aerogravity Data. Geophysical Research Letters 47, e2020GL088654, https://doi.org/10.1029/2020GL088654.

Greenbaum J.S., Blankenship D.D., Young D.A., Richter T.G., Roberts J.L., Aitken A.R.A., Legresy B., Schroeder D.M., Warner R.C., van Ommen T.D., Siegert M.J., 2015. Ocean access to a cavity beneath Totten Glacier in East Antarctica. Nature Geoscience 8, 294-298, https://doi.org/10.1038/ngeo2388.

Hodgson D.A., Jordan T.A., De Rydt J., Fretwell P.T., Seddon S.A., Becker D., Hogan K.A., Smith A.M., Vaughan D.G., 2019. Past and future dynamics of the Brunt Ice Shelf from seabed bathymetry and ice shelf geometry. The Cryosphere 13, 545-556, https://doi.org/10.5194/tc-13-545-2019.

Luyendyk B.P., Wilson D.S., Siddoway C.S., 2003. Eastern margin of the Ross Sea Rift in western Marie Byrd Land, Antarctica: Crustal structure and tectonic development. Geochemistry, Geophysics, Geosystems 4, https://doi.org/10.1029/2002GC000462.

Millan R., Rignot E., Bernier V., Morlighem M., Dutrieux P., 2017. Bathymetry of the Amundsen Sea Embayment sector of West Antarctica from Operation IceBridge gravity and other data. Geophysical Research Letters 44, 1360-1368, https://doi.org/10.1002/2016GL072071.

Millan R., St-Laurent P., Rignot E., Morlighem M., Mouginot J., Scheuchl B., 2020. Constraining an Ocean Model Under Getz Ice Shelf, Antarctica, Using A Gravity-Derived Bathymetry. Geophysical Research Letters 47, e2019GL086522, https://doi.org/10.1029/2019GL086522.

Tinto K.J., Padman L., Siddoway C.S., Springer S.R., Fricker H.A., Das I., Caratori Tontini F., Porter D.F., Frearson N.P., Howard S.L., Siegfried M.R., Mosbeux C., Becker M.K., Bertinato C., Boghosian A., Brady N., Burton B.L., Chu W., Cordero S.I., Dhakal T., Dong L., Gustafson C.D., Keeshin S., Locke C., Lockett A., O'Brien G., Spergel J.J., Starke S.E., Tankersley M., Wearing M.G., Bell R.E.,

2019. Ross Ice Shelf response to climate driven by the tectonic imprint on seafloor bathymetry. Nature Geoscience 12, 441-449, https://doi.org/10.1038/s41561-019-0370-2.
